# Outcomes of category III and I in immunocompetent patients of tuberculous lymphadenopathy treated in revised national tuberculosis control programme

**DOI:** 10.4103/0970-2113.68305

**Published:** 2010

**Authors:** Nageen Kumar Jain, Ashok Bajpai, Shikha Jain

**Affiliations:** *Department of Medicine, M.G.M. Medical College, Indore, India*

**Keywords:** DOTS, lymphadenopathy, tuberculosis

## Abstract

**Background:**

Retrospective observation analysis to evaluate the outcome of six month treatment regimen, CAT-III and CAT-I, for all patients diagnosed with tubercular lymphadenopathy at M.G.M. Medical College and M.Y. Hospital, Indore, India from January 2006 to December 2008.

**Materials and Methods:**

Out of 3158 cases of tuberculosis, there were 337 (10.67%) lymphnode cases; 31 (9.19%) HIV positive and 11(3.26 %) defaulters were excluded from the study. Of the remaining 295 cases of lymphadenopathy enrolled, 240 were on CAT-III and 55 cases of complicated lymphadenopathy were on CAT-I. All patients were followed for six months, to monitor response and complications. Patients with abscess formation or sinus formation underwent aspiration, AFB smear and culture sensitivity. In patients with persistent lymphadenopathy, repeat FNAC of the gland and in cases of hard lymphnode excision biopsy was done before receiving re-treatment regimen category –II or reserved drugs.

**Result:**

Out of 295 patients, 212 (71.86%) responded to treatment, however, 83 (28.14%) did not show response at the end of treatment. Of the 83 patients, 54 (65.06%) responded to re-treatment regimen CAT-II, while 29 (34.93%) were found to be drug resistant and were given second line anti tubercular drugs. In the follow-up at six months and later after the end of treatment, 11 (5.18%) relapse was seen.

**Conclusion:**

In our study, success rate of currently recommended regimens, category III and I under the revised national tuberculosis control programme in tubercular lymphadenopathy was 71.86%; with 28.34% of patients requiring re-treatment regimen. This requires a lager study involving more number of patients to prove adequacy of regimen.

## INTRODUCTION

A significant development in the treatment and control of tuberculosis has been the implementation of the Directly Observed Treatment short course (DOTS) along with fixed dose combination of existing drugs, since the WHO declaration of tuberculosis as a global emergency, in 1993. The National Tuberculosis Control Programm of India, operating since 1962 introduced DOTS for all patients in 1997.

The WHO recommendation of CAT-III in Tubercular Lymphadenopathy was based on paucibacillary nature of lymphadenopathy. At present, the effectiveness of intervention in controlling tuberculosis is determined by end of treatment outcomes and estimates of case detection. The global targets set for tuberculosis control are to detect 70% of new smear positive cases and successfully treat 85% of these cases. Mathematical modeling suggests that achievement of the target will precipitate an overall annual 6-7% reduction in tuberculosis incidence. If, however, disease persistence is substantial, at the end of treatment, target may be too low to bring about the predicted declines in incidence.

Despite DOTS being implemented for more than 10 years and millions of patients treated for tuberculosis, a few studies[1] have assessed the ability of standard DOTS regimens in tubercular lymphadenopathy to result in lasting cure for patients treated under routine programmatic conditions. This retrospective observational study aims to evaluate the efficacy of a six month regimen CAT-III and I in tubercular lymphadenopathy.

## MATERIALS AND METHODS

Revised national tuberculosis control programme (RNTCP) has recommended standardized six-month treatment for all new TB cases including two months initial phase of Isoniazid (H) Rifampicin (R) Pyrazinamide (Z) three times a week followed by a four-month continuation phase with H and R given three times a week. In cases of complicated Lymph node Ethambutol (E) is also added to the above regimen. This regimen is applied for all patients of tubercular lymphadenopathy. Patients were supervised while swallowing their drugs by the personnel of the health district, closest to their place of residence. At the end of the treatment, the number of doses taken for each patient was calculated. A tubercular lymphadenopathy case was defined as cured based on clinical criteria and if the patient had taken more than 90% of the doses.

The criteria for inclusion of patients in the present study were 1) Registration as a tubercular lymphadenopathy case 2) Treatment with 2 (HRZ)_3_ 4 (HR)_3_, 2 (HREZ)_3_ 4(HR)_3_. 3) Information available from the RNTCP database on the evaluation of the patient. Patients under 15 years of age, HIV positive and diabetes were excluded.

To assess, broad range of epidemiological information was collected for each case by reviewing the RNTCP data base, personal data, TB risk factors, diagnostic criteria, treatment received, follow up during treatment, side effects and complications during treatment and final patient outcome.

The diagnostic criteria to define a tubercular lymphadenopathy case are:

1. Clinical (Swelling, fever, loss of appetite and weight loss)
2. Bacteriological confirmation, and
3. Histological finding suggestive of TB (chronic granulomatous inflammation with or without caseating necrosis in tissue sample).

After the confirmation of diagnosis, all patients were followed every month for six months. Response to treatment was measured by

1. Improvement in clinical parameter
2. Average percentage reduction in the size of glands, which was measured vertical and horizontal by measuring scale at baseline and every month for six months.

Patients who missed doses or did not turn up for treatment when expected were actively traced as recommended by the RNTCP for all TB cases. If a patient defaulted for less than two months the same initial treatment was continued. However, if the patient defaulted for more than two months, an eight-month standard re-treatment CAT-II was offered.

After completion of treatment, patients were followed- up passively every month for six months to assess the relapse rate.

## RESULTS

A final total of 306 cases were included in the study, 121 (39.54%) men and 183 (59.80%) womenmale to female ratio 1:1.51 and a mean age of 25.2 years, with (Range15-65). Cervical lymph nodes were the most commonly affected; Eleven patients abandoned treatment and were excluded from the study. In all patients the tissue sample showed chronic granulomatous inflammation with or without caseous necrosis.

Of the 295 patients, 206 (70%) patients presented with low grade fever, 236 (80%) patients presented with loss of appetite and weight loss.

When we started treatment according to RNTCP guidelines, all patients were followed for six months to monitor response and complication. Out of 295, 212 patients showed significant reduction (<10 mm) in the size of gland within 16 weeks of starting treatment [Table 1]. Of the 295 patients, 67 (22.71%) developed abscess formation, pus was aspirated and same treatment was continued. Patients in whom repeat aspiration was serous in nature responded to the same treatment regimen. In cases where repeated aspiration was pus in nature and microscopic examination showed acid fast bacilli responded to DOT CAT-II. Second line antitubercular treatment was given when the case failed DOT CAT-II or AFB culture and sensitivity report showed resistance; 12 patients required extension of DOT category. In the follow-up conducted six months and later after the end of treatment, 11(5.18%) patients relapsed.

**Table 1:** Average percentage reduction in the size of gland according to duration in weeks (n=295)

Size of gland before starting T/t	4 wks	8 wks	12 wks	16 wks	20 wks	24 wks
100	59.24	75.05	89.79	96.49	98.24	98.24

All values are in percentage

Remaining 83 patients, when analyzed for treatment failure, some of the concomitant conditions were present [Table 2].

**Table 2:** Condition presented by 83 (28.13%) of 295 cases

Concomitant condition	No. of patients (n = %)
Family history of contact with known tuberculous patient	19 (29.89)
History of contact with known tuberculous patient	4 (4.81)
Alcohol / smoking	2 (2.40)
Complicated Tubercular lymphnode	
Multiple lymphnode	46 (55.42)
Abscess formation	67 (80.72)
Sinus formation	24 (28.91)
Mediastainal lymphnode with cervical lymphadenopathy	9 (10.84)
Abdominal lymphnode with cervical lymphadenopathy	4 (4.81)
Concomitant Pulmonary tuberculosis	18 (21.68)
Anemia	57 (68.6)

Some adverse drug reactions were also noticed during the course of treatment. These included nausea and vomiting in 98 (33.22%). None of the patients developed any treatment limiting adverse reaction [Table 3]

**Table 3:** Drug side effects in the 295 patients treated with CAT-I and III.

Side effects	n (%)
Any adverse reaction	110 (37.28)
Gastrointestine	98 (33.22)
Cutaneous	26 (8.81)
Arthralgia	4 (1.35)
Central Nervous System	12 (4.06)
Blood	1 (0.33)

## DISCUSSION

*Mycobacterium tuberculosis* infection in humans results in dissemination via lymphatic or hematogenous pathways or through contiguous spread to other organ or tissues. Although TB presents most commonly as pulmonary infection, between 10% and 20% of all patients present with extra pulmonary disease.[3,4] In our study, frequency of tubercular lymphadenopathy among all patients of tuberculosis was 10.67%. Because extrapulmonary tuberculosis re-present only 10-20% of TB infection, it is more difficult to diagnose and is not a public health threat because it is rarely transmittable, it has, therefore, never been a priority in TB control program.[5]

The mean age of patients was 25.5 years, tubercular lymphadenopathy is more common in female patients,[6–8] multiple lymph nodes and abscess formation, because it is more common during reproductive age group, hormonal changes may be responsible. In our study, 80% of patients presented with loss of appetite and weight loss. In other studies the prevalence of constitutional symptoms ranges from 33 to 85%.[9–11]

Among patients responded to CAT- III, showed more than 90% of reduction in the size of lymph node and serous fluid aspiration in cases of lymph node abscess within 16 weeks of starting treatment. In our study, percentage of complicated lymph node abscess and sinus formation was seen in 67 (22.71%) and 24(8.13%)[12] patients respectively.

Among patients who did not show response to treatment, either increase in the size of lymph node was seen or appearance of new lymph node and repeated aspiration was pus in nature. Eight patients required surgical excision because their lymph node was hard in consistency. Concomitant PTB and mediastinal lymph node were observed in 21.68% and 10.84% patients respectively among treatment failure cases.[13]

The defaulter rate in the present study was 3.26%.[14] None of the patients had drug- induced hepatitis; absence of side-effects is likely to have contributed to good treatment compliance.[15,16] In our study, success rate of category III and I treatment in tubercular lymphadenopathy was 71.86%. A study from Ajmer reported 63% response rate.[1] This warrants for a larger study involving patients of tubercular lymphadenopathy treated in RNTCP.
